# Interleukin-6 receptor antibodies (tocilizumab) in acute myocardial infarction with intermediate to high risk of cardiogenic shock development (DOBERMANN-T): study protocol for a double-blinded, placebo-controlled, single-center, randomized clinical trial

**DOI:** 10.1186/s13063-024-08573-0

**Published:** 2024-11-05

**Authors:** Joakim Bo Kunkel, Sarah Louise Duus Holle, Christian Hassager, Redi Pecini, Sebastian Wiberg, Pernille Palm, Lene Holmvang, Lia Evi Bang, Jesper Kjærgaard, Jakob Hartvig Thomsen, Thomas Engstrøm, Jacob Eifer Møller, Jacob Thomsen Lønborg, Martin Frydland, Helle Søholm

**Affiliations:** 1 Department of Cardiology, The Heart Centre, Copenhagen University Hospital – Rigshospitalet, Blegdamsvej 9, 2142, DK-2100 Copenhagen, Denmark; 2 Department of Clinical Medicine, Faculty of Health and Medical Sciences, University of Copenhagen, Copenhagen, Denmark; 3 Department of Cardiothoracic Anaesthesiology, The Heart Centre, Copenhagen University Hospital – Rigshospitalet, Copenhagen, Denmark; 4 Department of Cardiology, Bispebjerg Frederiksberg University Hospital, Copenhagen, Denmark; 5 Department of Cardiology, Odense University Hospital, Odense, Denmark; 6 Department of Cardiology, Zealand University Hospital, Roskilde, Denmark

**Keywords:** Acute myocardial infarction, Cardiogenic shock, Neurohormonal activation, ProBNP, Inflammation, Tocilizumab, IL-6RA, ORBI risk score, Percutaneous coronary intervention, Cardiac magnetic resonance imaging

## Abstract

**Background:**

Inflammation and neurohormonal activation play a significant role in the adverse outcome seen in acute myocardial infarction (AMI) and the development of cardiogenic shock (CS), which is associated with a mortality rate up to 50%. Treatment with anti-inflammatory drugs such as tocilizumab, an interleukin-6 receptor antagonist, has been shown to reduce troponin release and reduce the myocardial infarct size in AMI patients and it may therefore have cardioprotective properties.

**Methods:**

This is a double-blind, placebo-controlled, single-center randomized clinical trial, including adult AMI patients without CS at hospital arrival, undergoing percutaneous coronary intervention (PCI) within 24 h from symptom onset, and at intermediate to high risk of developing CS (ORBI risk score ≥ 10). A total of 100 participants will be randomized to receive a single intravenous dose of tocilizumab (280 mg) or placebo (normal saline). The primary outcome is peak plasma pro-B-type natriuretic peptide (proBNP) within 48 h, assessed using serial measurements at intervals: before infusion, 12, 24, 36, and 48 h after infusion. Secondary endpoints include the following: (1) cardiac magnetic resonance imaging (CMR) during 24–48 h after admission and at follow-up after 3 months with assessment of left ventricular area at risk, final infarct size, and the derived salvage index and (2) biochemical markers of inflammation (C-reactive protein and leukocyte counts) and cardiac injury (troponin T and creatinine kinase MB).

**Discussion:**

Modulation of interleukin-6-mediated inflammation in patients with AMI, treated with acute PCI, and at intermediate to high risk of in-hospital CS may lead to increased hemodynamic stability and reduced left ventricular infarct size, which will be assessed using blood biomarkers with proBNP as the primary outcome and inflammatory markers, troponin T, and CMR with myocardial salvage index as the secondary endpoints.

**Trial registration:**

Registered with the Regional Ethics Committee (H-21045751), EudraCT (2021–002028-19), ClinicalTrials.gov (NCT05350592). Study registration date: 2022-03-08, Universal Trial Number U1111-1277–8523.

## Administrative information

Note: the numbers in curly brackets in this protocol refer to the SPIRIT checklist item numbers. The order of the items was modified to group similar items (see http://www.equator-network.org/reporting-guidelines/spirit-2013-statement-defining-standard-protocol-items-for-clinical-trials/).

**Table:** 

Title {1}	Interleukin-6 Receptor Antibodies (tocilizumab) in Acute Myocardial Infarction with Intermediate to High Risk of Cardiogenic Shock Devel-opment (DOBERMANN-T): Study protocol for a double-blinded, placebo-controlled, single-center, randomized clinical trial
Trial registration {2a and 2b}.	Regional Ethics Committee: H-21045751. EudraCT: 2021–002028-19. ClinicalTrials.gov: NCT05350592. WHO Universal Trial Number: U1111-1277–8523
Protocol version {3}	Issue date: August 01, 2022. Protocol amendment number: 04.
Funding {4}	External financial grants: Novo Nordisk Foundation. Simon Spies Foundation. Helge Peetz og Verner Peetz og hustru Vilma Peetz Legat. Internal financial grants: Rigshospitalets Apparaturudvalg Rigshospitalets The Heart Centre Research Committee
Author details {5a}	Joakim Bo Kunkel, MD Department of Cardiology The Heart Centre Copenhagen University Hospital—Rigshospitalet Ryesgade 53, 9841, DK-2100 Copenhagen, Denmark E-mail: joakim.bo.kunkel@regionh.dk Phone: + 45 60 60 39 10
Name and contact information for the trial sponsor {5b}	Christian Hassager, MD, DMSc Department of Cardiology The Heart Centre Copenhagen University Hospital—Rigshospitalet Inge Lehmanns Vej 7, DK-2100 Copenhagen, Denmark E-mail: christian.hassager@regionh.dk. Phone: + 45 35 45 05 72
Role of sponsor {5c}	The Sponsor has status as Sponsor-Investigator and is thus directly involved with the initiation, design, and execution of the trial.

## Introduction

### Background and rationale {6a}

Acute myocardial infarction (AMI) remains a significant global health challenge despite advances in treatment leading to improved patient outcomes over recent decades. It is estimated that up to 10% of AMI patients experience acute hemodynamic compromise with cardiogenic shock (CS) development, which is associated with mortality rates exceeding 50% [1–3]. Approximately two thirds of CS patients present in advanced shock stages upon admission (the Society for Cardiovascular Angiography and Interventions (SCAI) shock stages C–E [2]), while the remaining one third of patients experience a decline in hemodynamic stability within hours to days following hospitalization [3, 4]. Recent research has highlighted the role of inflammation in both AMI and CS, although the exact mechanisms are not fully understood [5, 6].

Studies have linked systemic inflammatory responses with both the severity and the risk of CS onset [5, 7, 8]. Elevated levels of C-reactive protein (CRP), triggered by interleukin-6 (IL-6) signaling, correlate with mortality in patients with AMICS [9]. Tocilizumab, an IL-6 receptor antagonist (IL-6RA), has been shown to reduce IL-6 levels effectively, thereby suppressing CRP and exerting a systemic immunomodulatory effect [10]. This response has previously been associated with reduced troponin release and smaller infarct sizes in AMI patients [11, 12].

The systemic inflammatory markers inversely correlate with hemodynamic stability, including cardiac output, and have also been associated with mortality in patients with ST-segment elevation MI (STEMI) [13]. Elevated levels of pro-B-type natriuretic peptide (proBNP), indicating neurohormonal activation, have been observed in AMI patients who develop CS after hospitalization, compared to patients who remain hemodynamically stable [4].

The Observatoire Régional Breton sur L’Infarctus (ORBI) risk score, derived from clinical, hemodynamic, and procedural data collected during coronary angiography (CAG) and percutaneous coronary intervention (PCI), has shown high predictive accuracy for CS development in two French STEMI cohorts [14]. In this clinical trial, the ORBI risk score serves as the primary inclusion criterion, identifying patients who are initially stable but at high risk for hemodynamic deterioration. We hypothesize that modulation of interleukin-6-mediated inflammation with tocilizumab may lead to increased hemodynamic stability and reduced left ventricular (LV) infarct size by modulation of the inflammatory effect assessed by proBNP as a predictor for CS development in an AMI population treated with acute PCI and at intermediate to high risk of in-hospital CS.

## Objectives {7}

### Hypothesis

We hypothesize that treatment with tocilizumab in patients with AMI at intermediate high risk of in-hospital CS development will significantly reduce proBNP levels, indicating less hemodynamic compromise. Additionally, we hypothesize that treatment with tocilizumab will reduce the final infarct size and thus improve the LV myocardial salvage index.

#### Primary objectives

- To evaluate the effect of tocilizumab on change in peak proBNP within 48 h using blood samples taken before infusion and at 12, 24, 36, and 48 h after randomization.

#### Secondary objectives

- To evaluate the effect of tocilizumab on change in LV myocardial salvage using late gadolinium enhancement (LGE) CMR imaging during index admission (to establish area at risk) and at 3-month follow-up (to determine final infarct size)
- Change in CRP and leukocyte counts using blood samples taken before infusion and at 12, 24, 36, and 48 h after
- Change in troponin T and CKMB using blood samples taken before infusion and at 12, 24, 36, and 48 h after
- Change in proBNP at 3-month follow-up
- Change in 90-day survival rate

#### Trial design {8}

This single-center, investigator-initiated, placebo-controlled, double-blind, randomized phase II clinical trial involves patients with AMI who have undergone acute PCI (< 24 h from the first symptom). Upon admission and following screening in the catheterization laboratory (cath. lab.), patients are randomized 1:1 to receive either a 1-h single infusion of the interleukin 6 receptor blocker tocilizumab or placebo.

Additionally, patients in the trial are separately randomized in a 1:1 fashion to receive a 24-h infusion of low-dose dobutamine or placebo. It was predetermined that no interaction between tocilizumab and dobutamine was expected. Therefore, these two interventions are described as two separate trials, with the dobutamine intervention detailed in a simultaneously published manuscript. As part of an exploratory analysis, co-linearity between tocilizumab and dobutamine will be tested.

## Methods: participants, interventions, and outcomes

### Study setting {9}

The study takes place at Copenhagen University Hospital Rigshospitalet in Denmark, which is a public university hospital and tertiary heart center with 24/7 PCI services, to the eastern part of Denmark, serving approximately 2.7 million citizens out of an approximate total of 5.9 million in Denmark.

### Eligibility criteria {10}

Inclusion criteria:

- Suspected AMI < 24 h [15]
- Urgent revascularization of culprit lesion with PCI
- Presentation within 24 h of onset of chest pain
- ORBI risk score ≥ 10 [14]
- Age ≥ 18

Exclusion criteria:

- Unwilling to give informed consent to study participation
- Unable to give consent due to a language barrier
- Comatose after cardiac arrest
- CS with systolic blood pressure < 100 mmHg for more than 30 min or the need for vasopressors to maintain blood pressure and an arterial lactate level > 2.5 mmol/L prior to leaving the catheterization laboratory
- Other major clinical noncoronary conditions (stroke, sepsis, etc.), which can explain a high ORBI risk score
- Referral for acute coronary artery bypass grafting (CABG) (< 24 h) after CAG, whereas subacute (> 24 h will be included)
- Known tocilizumab allergy
- Pregnant or breastfeeding women
- Known liver disease/dysfunction
- Ongoing uncontrollable infection
- Immune deficiency/treatment with immunosuppressants
- Known, uncontrolled gastrointestinal disease predisposing to GI perforation

All screenings are registered with the purpose to report the reason for non-inclusion.

### Who will take informed consent? {26a}

This is not applicable. No identifying images or other personal or clinical details of participants are presented here or will be presented in reports of the trial results. The participant information materials and informed consent form are available from the corresponding author on request. Consent for participation in the study is obtained after oral and written information of the study by the co-investigator—a medical doctor or a senior medical student under supervision by a medical doctor. Patients have the option to involve an impartial witness when receiving information about the study. The patient is given 30 min with the opportunity to ask questions prior to giving consent. The time frame for consent has been determined based on urgency, as a longer delay to treatment implies a risk of CS prior to the initiation of the study drug. This approach aligns with the Declaration of Helsinki and adheres to relevant medical research legislation for consenting adult patients.

### Additional consent provisions for collection and use of participant data and biological specimens {26b}

Patients provide consent for collection and use of their data and biological specimens with the following permissions: to have blood samples stored in a biobank for future measurements of biomarkers related to inflammation, neurohormonal activation, neuronal injury, connective tissue function, and other relevant pathophysiological processes. Any remaining biological material from the research biobank will be transferred to and stored for up to 10 years in a separate biobank exclusively for future research purposes. Patients retain the right to request the destruction of their biological specimens, in accordance with national regulations.

## Interventions

### Explanation for the choice of comparators {6b}

To modulate the inflammatory response, which is suggested associated with hemodynamic instability, troponin leakage, and LV infarct size, a single 280 mg dose of the tocilizumab was selected [10, 11]. This dosage falls within the standard range for adults with approved indications, e.g., rheumatoid arthritis.

### Intervention description {11a}

Patients allocated to the active group will undergo a 1-h intravenous infusion, receiving a fixed dose of 280 mg of tocilizumab. This dose is prepared as a fixed-dose formulation in isotonic saline at a rate of 100 mL per h, leading to a total volume of 100 mL. Patients in the placebo group will receive a 1-h infusion of isotonic saline at the same rate of 100 mL per h.

All infusions are prepared on an individualized basis for each patient by a collaborating cardiac unit at the center. This approach ensures that blinding is maintained for the treating unit, including clinical staff, patients, investigators, and sponsor. Each infusion kit is uniquely labeled ensure safe and accurate administration. The assigned treatment arm will not be visually or otherwise discernible. Infusions will be carried out through either a central or peripheral IV therapy line using standard infusion pumps. The administration of infusions will start as early as possible after obtaining informed consent and no later than 2 h after the acute PCI procedure.

### Criteria for discontinuing or modifying allocated interventions {11b}

Patients have the right to withdraw from the study at any time and for any reason, without any impact on their future medical care. This withdrawal will be done in accordance with relevant legislation and ethical guidelines. Data collected up to the date of withdrawal will be retained. Patients who withdraw may still be offered the option to continue sampling in a reduced form subject to individual agreement. In certain clinical situations, the sponsor and/or the investigator may decide to withdraw a patient from the study. This decision can be made if termination is in the best medical interest of the patient.

An end-of-study case report form will be completed to document the final termination in the study.

The infusion rate and duration will generally not be subject to modification. However, if a suspected unexpected serious adverse reaction (SUSAR) occurs during infusion, the infusion will be promptly stopped, and treatment will be initiated following applicable clinical guidelines.

### Strategies to improve adherence to interventions {11c}

The study has established standard operating procedures (SOPs) to standardize procedures and clinical personnel receive role-specific training. An investigator is available at all times of day, ensuring access to support. Infusions are administered only once and simultaneously by the investigator, minimizing errors.

### Relevant concomitant care permitted or prohibited during the trial {11d}

Screening is ongoing during the acute PCI. The infusion is administered on top of current standard care protocols followed in the Cardiac Care Unit after PCI. No delay in treatment is expected, and no restrictions apply to standard concomitant care or necessary interventions.

### Provisions for posttrial care {30}

Participants in the study receive coverage provided by the Danish Patient Compensation Scheme, as do all individuals receiving healthcare services under the Danish Patient Compensation Act. Following the concluding follow-up appointment, the team will evaluate any clinical signs or symptoms reported by patients, indicating the onset or exacerbation of conditions (this includes mental health issues and depression) that have not been previously diagnosed or treated. Where required, patients will be referred for additional evaluation and care.

### Outcomes {12}

#### Primary endpoint

The primary endpoint is the between-group difference (active/placebo) in peak proBNP within 48 h from randomization, Table 1.

**Table 1:** Summary

**Timepoint**	Enrolment	Post-allocation * denotes 3-h intervals												Follow-up
	**Before infusion**	**1 h**	**2 h**	**3 h**	**6 h**	*	**12 h**	*	**24 h**	*	**36** **h**	*	**48 h**	**3 months**
**Enrolment**														
Eligibility screen	X													
Informed consent	X													
Randomization	X													
**Interventions**														
Tocilizumab 280 mg/placebo		X												
**Assessments**														
Arterial puncture (lactate, blood glucose)	X													
proBNP, biomarkers, biobank	X						X		X		X		X	X
CMR													**X**	**X**
TTE	X												X	X
12-lead ECG	X						X		X		X		X	
Telemetry	X													
BP, P, RR, SAT, Tp	X	X	X	X	X	X	X	X	X	X	X	X	X	
QoL, MoCA, GS, frailty													X	X

*Abbreviations:* *h* Hour, *proBNP* Pro-B-type natriuretic peptide, *CMR* Cardiac magnetic resonance, *TTE* Transthoracic echocardiography, *ECG* Electrocardiography, *BP* Blood Pressure, *P* Pulse, *RR* Respiratory Rate, *Tp* Temperature, *QoL* Quality of life, *MoCA* Montreal Cognitive Assessment, *GS* Grip strength

#### Secondary endpoints

Infarct characteristics.

- Between-group difference (active/placebo) in myocardial salvage using contrast-enhanced CMR imaging performed during index admission and at 3-month follow-up
- Between-group difference (active/placebo) in troponin T and creatine kinase MB (CKMB)) over time using blood samples taken before infusion and at 12, 24, 36, and 48 h after randomization as well as at 3-month follow-up

Inflammation.

- Between-group difference (active/placebo) in CRP and leukocyte counts, over time using blood samples taken at baseline, before infusion and at 12, 24, 36, and 48 h after infusion as well as at 3-month follow-up.

### Participant timeline {13}

### Sample size {14}

The sample size calculation is based on recent, unpublished data on atrial natriuretic peptide (ANP) levels in STEMI patients [16]. Assuming a log-normal distribution with a variation coefficient of 0.59 for proBNP levels (similar to ANP), an alpha level of 0.05, and a power of 0.86, a total of 88 patients would be needed to detect a 30% reduction in proBNP from a baseline of 1338 ng/L to 937 ng/L [17]. To account for potential dropouts and missing data, total of 100 participants will be enrolled in the trial.

### Recruitment {15}

The recruitment period is estimated as three years based on retrospective analysis of the frequencies of ORBI risk score distribution among STEMI patients taken the inclusion and exclusion criteria intro account, utilizing data from prior studies conducted at the center [8].

## Assignment of interventions: allocation

### Sequence generation {16a}

This study is designed to investigate the effect of the study drugs versus placebo. Allocation is based on a computerized pseudorandom number generator with a randomly chosen seed. Block sizes are predefined to ensure balanced group distribution, with the sequence details kept confidential from investigators to preserve allocation integrity and prevent bias.

### Concealment mechanism {16b}

Allocation concealment is ensured using nonsequential alphanumerical randomization keys. Each key, tied to specific block variables, is securely stored in a dedicated module of the electronic case report form (eCRF) system.

### Implementation {16c}

For each enrolled participant, the investigator retrieves the unique randomization key from the eCRF. This key, combined with the participant ID and weight, is forwarded to the drug-preparing unit for blinded infusion kit preparation. The drug preparation team uses the randomization key in a secure system to unveil the assigned intervention momentarily. After preparing the study drug, the procedure is documented in an unblinded eCRF section, isolated from investigator access during the trial, maintaining blinding integrity unless protocol exceptions necessitate access.

## Assignment of interventions: blinding

### Who will be blinded {17a}

The study is double-blinded, covering the sponsor, investigators, patients, and clinical staff in the admission department after the PCI. Alphanumerical randomization keys, devoid of treatment details, alongside uniformly labeled and visually identical infusion kits, ensure the concealment of treatment assignments.

### Procedure for unblinding if needed {17b}

The trial will maintain double blinding throughout the study duration whenever possible. However, a controlled unblinding process is in place for specific situations:

- Emergencies: if a participant experiences a serious adverse event and the treating physician needs to know the allocated treatment (active or placebo) to guide further management
- Audits or data verification: authorized personnel from regulatory bodies or the study sponsor may require unblinding to verify trial conduct and data integrity

To ensure controlled access to treatment allocation information and maintain blinding integrity as much as possible, the following procedures are implemented.

## Data collection and management

### Plans for assessment and collection of outcomes {18a}

Data collection will be performed by trained personnel familiar with the study protocol to ensure accuracy and consistency. Vital signs and clinical data will be collected from electronic medical records. Biochemical test results will be obtained from the Department of Clinical Biochemistry, with validation records maintained by the authors for transparency. Data collection forms will be primarily electronic (eCRF) but will also be available in paper format for backup, with originals archived. Imaging data will be stored in their original formats within a secure clinical imaging archive. Blinding will be maintained whenever possible during image acquisition, analysis, and interpretation by investigators. Interobserver variability will be assessed to ensure data reliability.

### Plans to promote participant retention and complete follow-up {18b}

Infusions are completed during the initial admission, with 24/7 access to investigators for assistance. At discharge, patients are assigned a trial representative for any follow-up queries. Follow-up visits are scheduled flexibly, considering patients’ transportation and assistance needs.

### Data management {19}

Trial data are recorded using the eCRF REDCap hosted by the Capital Region of Denmark. This configuration adheres to both scientific and regulatory criteria for data entry, coding, security, and storage, with prior approval at the center for these specific purposes [18, 19].

### Confidentiality {27}

Personally identifiable information is securely maintained within approved systems at the center, encompassing electronic health records and related databases that encompass laboratory analyses and imaging studies. Data management complies with the European General Data Protection Regulation (GDPR) to protect participant confidentiality and privacy.

## Plans for collection, laboratory evaluation, and storage of biological specimens for genetic or molecular analysis in this trial/future use {33}

### Blood sample collection

Timepoints: Baseline, before infusion and at 12, 24, 36, and 48 h after infusion, and at a 3-month follow-up visit. Tubes: Blood will be collected in tubes containing ethylenediaminetetraacetic acid (EDTA), lithium-heparin, sodium citrate, and serum. Processing: Samples will be centrifuged at 2000 g relative centrifugal force for 10 min at 20–24 °C. Storage: Samples will be stored at − 80 °C.

### Clinical assays

Clinical assays will be performed by the center Department of Clinical Biochemistry and reported within the electronic health records system. The sampling procedure is as follows: venous blood sampling will be performed according to a standard operating procedure (SOP).

### Biobank samples

Biobank samples will be collected and stored in a biobank by the Department of Clinical Biochemistry in accordance with a cooperation agreement.

## Statistical methods

### Statistical methods for primary and secondary outcomes {20a}

Categorical data will be presented as frequencies (number of patients), while continuous data will be presented as mean and standard deviation (SD) if normally distributed or median and interquartile range (IQR, 25th–75th percentile) if not normally distributed. Both *p*-values and confidence intervals will be reported where appropriate.

Categorical outcomes will be analyzed using chi-squared tests or Fisher’s exact tests, depending on data characteristics. Continuous outcomes will be analyzed using appropriate parametric or non-parametric tests based on their distribution.

The primary outcome peak proBNP will be log-transformed and analyzed using baseline correction (measurement before infusion) with the analyses of covariance (ANCOVA) models with adjustment for ORBI risk score and the effect of the intervention in concurrent trial (*DOBERMANN-D*). Interaction analyses will be performed to assess the potential interaction between the two intervention groups. These models will also be applied for the secondary endpoints of biomarkers and imaging parameters.

The alpha level for statistical significance is set at 0.05. To account for multiple comparisons and reduce the risk of type I error, Bonferroni correction will be applied to adjust the significance levels for repeated testing across the secondary outcomes.

All data analyses will be conducted using the latest stable version of R statistical software, and the version used will be reported in the final manuscript.

### Interim analyses {21b}

No interim analyses are planned for this study.

### Methods for additional analyses (e.g., subgroup analyses) {20b}

To assess for any interaction with the other intervention (dobutamine vs. placebo) in the trial, subgroup analyses according to the assigned treatment will be performed (*DOBERMANN-D*).

### Methods in analysis to handle protocol nonadherence and any statistical methods to handle missing data {20c}

Analyses will be performed on the modified intention-to-treat population. Missingness will be evaluated, and if above 5%, imputation will be applied.

### Plans to give access to the full protocol, participant-level data, and statistical code {31c}

A completely deidentified dataset will be made available upon reasonable request.

## Oversight and monitoring

### Composition of the coordinating center and trial steering committee {5d}

The study is a single-center study, and thus day-to-day coordination efforts are overseen by the sponsor and primary investigator in a collaboration between coinvestigators and other study personnel. All of these have experience in conducting clinical trials in similar patient populations. Meetings are held as needed to address operational issues and assess the progression of the trial. There is no trial steering committee with independent members or a stakeholder and public involvement group (SPIG) in the current study.

### Composition of the data monitoring committee, its role and reporting structure {21a}

A data monitoring committee has not been established. An audit scheme is implemented, which includes unblinded monitoring of the assigned intervention and quality of the data generated for primary endpoint analyses (see 23).

### Adverse event reporting and harms {22}

Serious adverse events (SAEs) and suspected unexpected serious adverse reactions (SUSARs) are systematically documented and assessed. For each SAE and SUSAR, the sponsor completes an adverse event form detailing the event description, timing, severity, relationship to the intervention, actions taken, and outcome. Participants experiencing any adverse events are managed according to standard protocols, with follow-up continuing until resolution or stabilization. Annually, a safety report summarizing SAEs and reactions is submitted to the Danish Health and Medicines Authority.

### Frequency and plans for auditing trial conduct {23}

The GCP Unit of The Capital Region of Denmark oversees audits of trial conduct, adhering to the standards set by The International Council for Harmonization of Technical Requirements for Pharmaceuticals for Human Use (ICH), including a prior GCP agreement that encompasses risk analysis. These audits are conducted independently and involve direct observation of how patients are allocated to treatments and how investigational medicinal products (IMPs) are administered. After each audit, which occurs every 3 to 6 months, reports are provided to the sponsor. Each audit aims to thoroughly review the collected data from at least 10% of the patients who have enrolled in the trial.

### Plans for communicating important protocol amendments to relevant parties (e.g., trial participants, ethical committees) {25}

A digital workflow ensures all relevant parties, including trial participants and ethical committees, are promptly informed of any protocol amendments. Amendments are enacted only after obtaining regulatory approval.

### Dissemination plans {31a}

The trial findings will be prepared for publication in internationally recognized peer-reviewed journals and presented at international conferences. During the consent process, patients and their next of kin will be offered the option to receive the trial results upon completion.

## Discussion

**Fig. 1:**
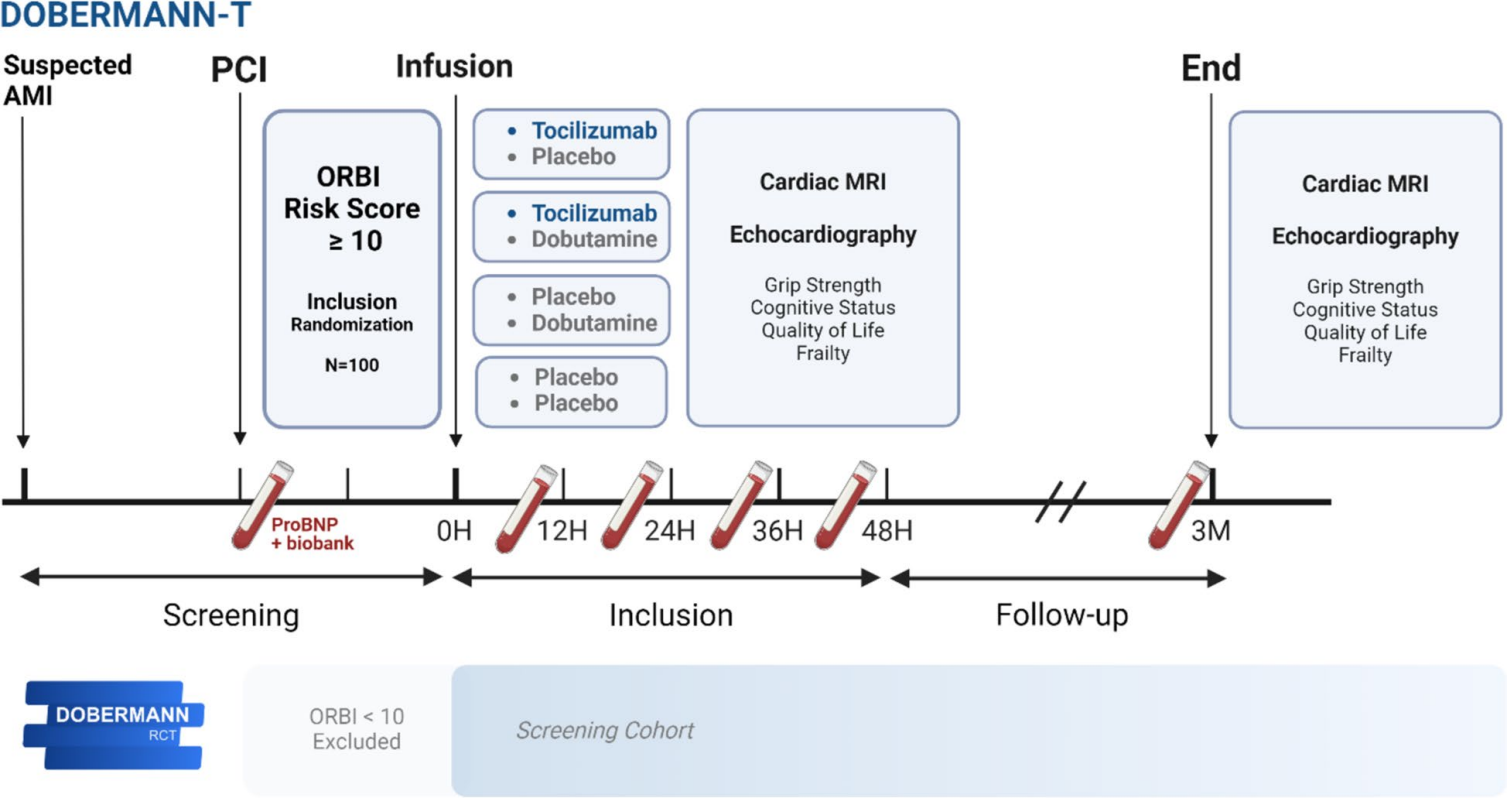
Flowchart of the DOBERMANN-T trial—from screening to follow-up. Details concerning the dobutamine/placebo intervention group are published separately (DOBERMANN-D). Abbreviations: PCI, percutaneous coronary intervention; ORBI, Observatoire Régional Breton sur l’Infarctus du myocarde; MRI, magnetic resonance imaging; H, hours

**Fig. 2:**
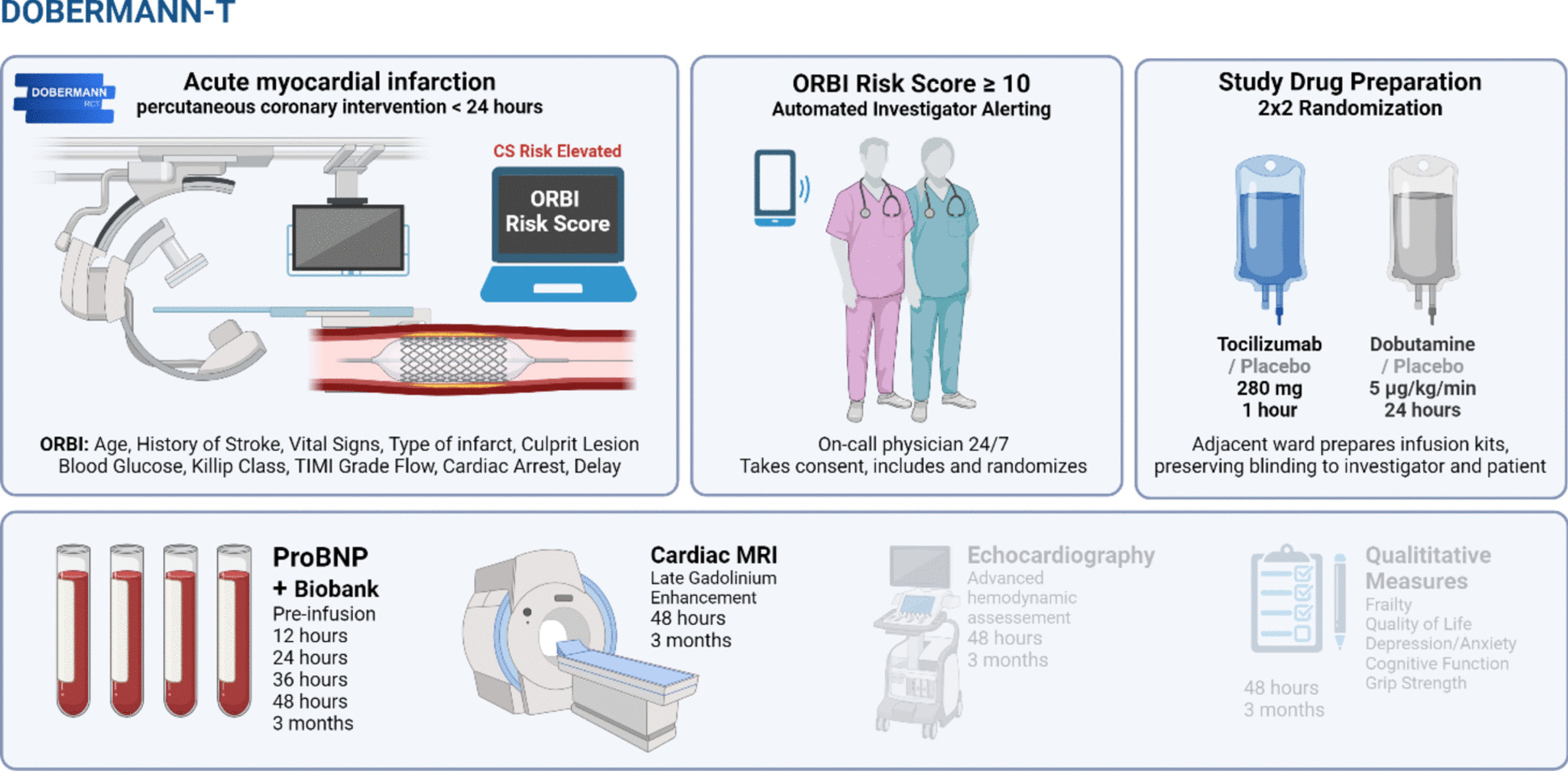
Study concept, interventions and endpoints. Abbreviations: CS, cardiogenic shock; ORBI, Observatoire Régional Breton sur l’Infarctus du myocarde; TIMI, thrombolysis in myocardial infarction; ProBNP, pro B-type natriuretic peptide; MRI, magnetic resonance imaging

The DOBERMANN-T trial investigates the anti-inflammatory effects of the IL-6RA tocilizumab on the risk of CS development in patients with AMI who are at intermediate to high risk (Figs. 1 and 2). The study is a randomized, double-blinded trial with several objectives: firstly, to identify at-risk patients using the ORBI risk score [14]; secondly, to administer tocilizumab after acute PCI to modulate the systemic inflammatory responses potentially contributing to CS development; and thirdly, to assess the effect of tocilizumab on myocardial area at risk, final infarct size, and salvage index assessed by CMR.

The use of the ORBI clinical risk score allows for early risk assessment during PCI by identifying patients who, despite appearing hemodynamically stable, show early signs of potential CS development [4]. ProBNP, a marker released in response to myocardial stretch [20] and ischemia [21–23], is used as the primary endpoint, as it reflects the degree of hemodynamic deterioration. Plasma concentration of proBNP is inversely correlated with hemodynamic status, including cardiac output [24], and a rise has been associated with increased mortality in patients with STEMI [25]. This trial aims to determine whether administration of tocilizumab is able to reduce proBNP levels in the hours following PCI suggesting a more stable hemodynamic situation.

Tocilizumab has shown potential for reducing myocardial injury in adjacent populations. The IMICA trial included comatose patients resuscitated from out-of-hospital cardiac arrest and showed that tocilizumab significantly reduced the systemic inflammatory markers CRP and leukocyte counts as well as markers of myocardial injury such as CKMB and troponin levels [10]. Notably, the trial recorded a marked decrease in N-terminal proBNP levels. This supports the hypothesis that reducing inflammation could mitigate myocardial injury after AMI, thereby potentially decreasing the progression of CS.

Meanwhile, the ASSAIL trial provided evidence addressing the efficacy of tocilizumab administered prior to primary PCI in patients with STEMI [12]. The trial found that tocilizumab improved myocardial salvage as measured by CMR, indicating less myocardial damage and better functional recovery. This outcome supports an effect on myocardial recovery after coronary reperfusion. This aligns with previous research, showing that IL-6 pathways might directly influence LV remodeling [26].

Tocilizumab, like other immunomodulatory agents, may pose risks such as increased infection susceptibility. However, its established use in rheumatoid arthritis and severe COVID-19 suggests it is generally safe, even in critically ill states, as demonstrated in recent trials [27].

Reduction of the systemic inflammation in patients with AMI, treated with acute PCI, and at intermediate-high risk of in-hospital CS may lead to reduction of the myocardial left ventricular infarct size and hemodynamic stability, which will be assessed using blood biomarkers with peak proBNP as the primary outcome and CMR within the initial 48 h and repeated at 3-month follow-up.

In conclusion, should the anti-inflammatory drug tocilizumab prove effective in lowering proBNP levels and improving myocardial salvage, it would support the hypothesis that early immunomodulatory intervention can benefit AMI patients at risk of developing CS.

## Trial status

Protocol version: 2.7.6 (revision 4 from approval), 14 November 2022.

Recruitment began on 1 March 2022 and is commencing as planned, and the last patient will estimated be included by December 2024. Data will be available after contact to the corresponding author.

## Data Availability

All principal investigators will have full access to the final trial data set. Project data will be stored in a secured repository with appropriate access control measures. Data will be made further available upon reasonable request at the discretion of the investigators.

## Abbreviations

AE: Adverse event
ALT: Alanine transaminase
AMI: Acute myocardial infarction
ANP: Atrial natriuretic peptide
BP: Blood pressure
CABG: Coronary artery bypass grafting
CAG: Coronary angiography
Cath lab: Catheterization laboratory
CCU: Cardiac care unit
CFS: Clinical Frailty Scale
CICU: Cardiac intensive care unit
CMR: Cardiac magnetic resonance
CRP: C-reactive protein
CS: Cardiogenic shock
eCRF: Electronic case report form
EDTA: Ethylenediaminetetraacetic acid
EQ-5D-5 L: 5-level EuroQol-5
GCP: Good Clinical Practice
GCS: Glasgow Coma Scale
GI: gastrointestinal
GS: Grip strength
H: Hours
HADS: Hospital Anxiety and Depression Scale
ICH: International Council for Harmonization of Technical Requirements for Pharmaceuticals for Human Use
IHD: Ischemic heart disease
IL-6RA: Interleukin-6 receptor antibody
IMP: Investigational Medicinal Products
LVEF: left ventricular ejection fraction
MoCA: Montreal Cognitive Assessment
OHCA: Out-of-hospital cardiac arrest
ORBI: Observatoire Régional Breton sur l’Infarctus
P: Pulse rate
PCI: Percutaneous coronary intervention
proBNP: Pro-B-type natriuretic peptide
QoL: Quality of life
REDCap: Research Electronic Data Capture
RR: Respiratory rate
SAT: Saturation
SAE: Serious adverse event
SCAI: Society for Cardiovascular Angiography and Interventions
SD: Standard deviation
SIRS: Systemic inflammatory response
SOFA: Sequential organ failure assessment
STEMI: ST-elevation myocardial infarction
SUSAR: Serious adverse reaction
T: Temperature
TTE: Transthoracic echocardiography
VTI: Velocity Time Integral
